# The Detection of Cheating on E-Exams in Higher Education—The Performance of Several Old and Some New Indicators

**DOI:** 10.3389/fpsyg.2020.568825

**Published:** 2020-10-02

**Authors:** Jochen Ranger, Nico Schmidt, Anett Wolgast

**Affiliations:** ^1^Department of Psychology, Martin-Luther-University Halle-Wittenberg, Halle (Saale), Germany; ^2^Department of Psychology, University of Applied Sciences Hannover, Hanover, Germany

**Keywords:** cheating (education), classification and regression tree (CART), person fit, response time, higher education

## Abstract

In this paper, we compare the performance of 18 indicators of cheating on e-exams in higher education. Basis of the study was a field experiment. The experimental setting was a computer assisted mock exam in an introductory course on psychology conducted at a university. The experimental manipulation consisted in inducing two forms of cheating (pre-knowledge, test collusion) in a subgroup of the examinees. As indicators of cheating, we consider well-established person-fit indices (e.g., the U3 statistic), but also several new ones based on process data (e.g., response times). The indicators were evaluated with respect to their capability to separate the subgroup of the cheaters from the remaining examinees. We additionally employed a classification tree for detecting the induced cheating behavior. With this proceeding, we aimed at investigating the detectability of cheating in the day-to-day educational setting where conditions are suboptimal (e.g., tests with low psychometric quality are used). The indicators based on the number of response revisions and the response times were capable to indicate the examinees who cheated. The classification tree achieved an accuracy of 0.95 (sensitivity: 0.42/specificity: 0.99). In the study, the number of revisions was the most important predictor of cheating. We additionally explored the performance of the indicators to predict the specific form of cheating. The specific form was identified with an accuracy of 0.93.

## 1. Introduction

In educational testing and academic examination, the term cheating is used to denote all forms of illegitimate activities that are aimed at increasing one's test performance. These activities comprise using unauthorized materials (e.g., calculators), resorting to additional information during an exam (e.g., via cheat sheets), answer copying, collusion among examinees, the acquisition of test questions (aka pre-knowledge) or having another person take the test instead of oneself (Bernardi et al., 2008). Cheating affects the validity of examination in higher education and impairs all decisions that are based on test results. Cheating is frequent among students at high-schools and universities. In the USA, about 50% of all high school students reported having cheated on an exam at least once in the last year (e.g., Cizek and Wollack, 2017a; Meiseberg et al., 2017) and about 10% of all university students admitted having copied from other examinees (e.g., McCabe, 2016). Given the high prevalence of cheating, it is no surprise that the causes, the detection and the prevention of cheating have been investigated intensively. Three recent published monographs address the detection of cheating on tests (Wollack and Fremer, 2013; Kingston and Clark, 2014; Cizek and Wollack, 2017b).

In this paper, we focus on cheating on e-exams in higher education. With e-exams in higher education we denote all forms of computer assisted, curricular tests used in colleges or universities for individual performance assessment. This definition excludes large-scale tests like TOEFL iBT (Alderson, 2009) or GRE (ETS, 2012). From a psychometric point of view, the detection of cheating on e-exams in higher education poses several challenges: (1) The exams are composed with regards to content-related criteria. They are usually criterion-referenced and consist of heterogeneous questions covering different areas. Their psychometric properties are also not investigated in a pretest. Ordinary exams might therefore be measurement instruments that meet the standard assumptions of test theory (monotone item characteristic functions, unidimensionality and local independence) to a lesser extent than the professional tests used in large-scale assessment. This complicates the detection of cheating as most indices of person fit require data with a clear unidimensional dominance structure. (2) Exams in higher education are often administered in lecture halls or classrooms. In this setting, there is little control over the test context. Examinees, for example, might be allowed to interrupt the exam shortly. There is often no fixed question sequence and previous responses can be revised. Process data recorded during the exam may have a high variability which weakens the relation to the response process. (3) The number of examinees is much lower in e-exams than in large-scale assessment. This complicates the analysis, as standard techniques, such as conditioning on subgroups with the same test score are not feasible in small samples. Fitting a latent trait model is also not possible. Against this background, it is unclear whether the findings on the detection of cheating in large-scale assessment are transferable to e-exams in higher education.

In this paper, we investigate the performance of 18 indicators to detect two forms of cheating, namely pre-knowledge and test collusion. We considered well-established person-fit indices (e.g., the U3 statistic proposed by van der Flier, 1977), but also new ones based on process data. Basis of the investigation was a mock exam in an introductory lecture on psychology. The exam was computer assisted and consisted of questions with a multiple choice response format. Examinees were not restricted with regard to the order and the frequency they worked on the single questions. With this implementation we intended to allow the examinees a similar control over their proceeding as in a paper and pencil test; note that a similar design was used by the OECD for the Pisa-based test for schools (Wise and Gao, 2017). For each examinee, the responses were recorded, but also process data like the response times, the number of response revisions, and the sequence in which the questions were answered. These data are usually contained in the log-files recorded by a computer based testing system (von Davier et al., 2019). The detection of cheating was investigated by means of a field experiment. A subgroup of the examinees was induced to cheat, either by providing pre-knowledge or by provoking test collusion. The focus of the study was on the performance of the indicators to detect these cheaters. The paper is organized as follows. First, we give a brief review on the indicators of cheating. Then, we describe the study and the approach to data analysis. Finally, we compare the discriminatory power of all indicators and determine the detection rate that can be achieved by a classification tree when all indicators are used.

## 2. Indicators of Cheating

The literature on the detection of cheating is comprehensive. Indicators of cheating can be distinguished whether they are based on a specific latent trait model or not; whether they are designed to detect a specific form of cheating or intended to indicate misfit in general; whether they require a hypothesis about the affected questions/students or not; or whether they are based on the responses or on process data like the response times or the number of response revisions. For sake of brevity, we will not try to give an exhaustive overview of the topic here, but refer to the monographs of Kingston and Clark (2014) and Cizek and Wollack (2017b) and to the overviews of He et al. (2018) and Meijer and Sijtsma (2001) instead. The present study is limited to indicators of cheating that are generally applicable and easy to calculate. Indicators that are based on an item response model are, for example, excluded. Indicators that depend on additional aspects like the students' performance in the past, item parameter estimates from pretest samples or specific hypotheses about the cheaters are also not considered.

In the paper, we considered five different types of indicators. We considered indices of person-fit that evaluate the regularity of an examinee's response pattern. These indices were the U1 statistic (van der Flier, 1977), the U3 statistic (van der Flier, 1982), the CS statistic (Sato, 1975), and the H^T^ statistic (Sijtsma, 1986). We also considered indicators that are based on the response times. These indicators were the KL statistic (Man et al., 2018), a Z^2^ statistic similar to the one proposed by Marianti et al. (2014) and Sinharay (2018), and a new index—the KT statistic—that evaluates the Guttman homogeneity of an examinee's response time pattern. We furthermore considered indicators that were based on the number of response revisions and the corresponding response times (Qualls, 2005; Bishop and Egan, 2017). Among these indicator were two indicators (N1, N2) that were related to the number of response revisions, two indicators (NC1, NC2) that were related to the number of wrong-to-right revisions and three indicators (T1, T2, T3) that were related to the revision times. We additionally considered an indicator that was based on the relation between the responses and the response times. This indicator (CD) was Cook's distance from the regression of the total testing time on the sum score of the responses. We finally considered two indicators that were based on the similarity of response patterns from different examinees (Maynes, 2017; Zopluoglu, 2017). The first indicator (PT) was formed on basis of the number of identical responses and the second indicator (PI) on basis of the number of identical incorrect responses; for earlier studies on the performance of some of these indicators in the field of educational testing, see Karabatsos (2003), Tendeiro and Meijer (2014), Kim et al. (2017), Sinharay (2017), and Man et al. (2018). In the following, we will briefly review the employed indicators. Formulas specifying the calculation of the indicators can be found in Supplementary Material.

As the implementation of the e-exam did not restrict the frequency with which an examinee could access a question, we distinguish between an attempt and a revision. With attempt we refer to any distinct period of time in which the question content appears on an examinee's screen irrespective of the response that is given. With revision, we refer to particular attempts in which the response was changed. The indicators based on the responses were calculated with the first or the final response depending on whether an examinee changed his/her response to a question. The indicators based on the response times were calculated with the total testing times an examinee spent on the single questions during all attempts. The remaining indicators are calculated on basis of the responses and the response times in the last attempts or the last revisions, depending on the specific variant.

### 2.1. Indicators Based on Responses

#### 2.1.1. U1 Statistic

The U1 statistic of van der Flier (1977) assesses how Guttman homogeneous a response pattern is. A response pattern is denoted as Guttman homogeneous in case solving a question implies that all easier questions have been solved as well. Guttman homogeneous response patterns are the ideal for unidimensional scales with a pronounced dominance structure. The U1 statistic compares the actual number of Guttman errors in a response pattern to the maximal number that could have occurred. A value of zero indicates perfect Guttman homogeneity, a value of one the contrary. Large values of the U1 statistic are usually understood as being indicative of an irregular response process.

#### 2.1.2. U3 Statistic

The U3 statistic of van der Flier (1982) is also based on the number of Guttman errors. In contrast to the U1 statistic, the U3 weights the Guttman errors. Errors in very easy and very difficult questions are less probable and receive more weight. The U3 statistic is zero in case a response pattern is perfectly Guttman homogenous. A value of one indicates the contrary. Large values of the U3 statistic are usually considered as being indicative of an irregular response process.

#### 2.1.3. CS Statistic

The CS statistic of Sato (1975) is a further measure of the Guttman homogeneity of a response pattern. It is based on the covariance of the responses in a response pattern with the solution probabilities of the corresponding questions. This covariance is compared to the covariance a perfect Guttman pattern would have. The CS statistic is similar to the U3 statistic. It is zero in case a response pattern is perfectly Guttman homogeneous. It has, in contrast to the U1 and U3 statistic, no fixed upper bound. Large values of the CS statistic are supposed to indicate data irregularities.

#### 2.1.4. H^T^ Statistic

The H^T^ statistic proposed by Sijtsma (1986) assesses how similar a response pattern is to the remaining response pattern in the sample. The similarity is evaluated via the covariances between the responses of the response pattern and the responses of the remaining response pattern. The covariances are averaged and compared to the average of the maximal covariances that could have been achieved for the observed test scores of the examinees. In contrast to the statistics that assess the Guttman homogeneity, the H^T^ statistic does neither require unidimensionality nor a dominance structure. The H^T^ statistic is zero when the average covariance between an examinee's responses and all other responses is zero. Its maximal value is one. Small values of H^T^ are characteristic of data anomalies.

### 2.2. Indicators Based on Response Times

#### 2.2.1. KL Statistic

The KL statistic proposed by Man et al. (2018) evaluates the congruence of an examinee's response time profile with the average response time profile in the sample. It is based on the proportions of the total testing time an examinee spends on the single questions. The individual proportions are compared with the proportions the question specific response time averages make up of the average total testing time. The congruency of the two vectors of proportions is assessed via the Kullback-Leibler divergence. The divergence is zero in case all corresponding proportions are identical. An unusual distribution of the total testing time over the questions inflates the statistic. Large values are thus assumed to be indicative of cheating.

#### 2.2.2. KT Statistic

The KT statistic assesses whether the individual response times of an examinee have the same order as the typical response times in the sample. It is defined as Kendall's tau correlation (Kendall, 1938) between the response times of an examinee and the median response times in the questions. In this aspect, the statistic is analog to the statistics that assess the Guttman homogeneity of the responses. Using the tau correlation and the median response time increases the robustness of the approach. It also reduces the influence of possible response time transformations, as, for example, the log-transformation. Such transformations affect the KL statistic, but not the KT statistic. The KT statistic is one in case the order of the individual response times and the order of the medians is identical. Low values are indicative of an irregular way of responding.

#### 2.2.3. Z^2^ Statistic

The Z^2^ statistic assesses whether some response times of an examinee are outlying. The variant that we use in this manuscript is based on the doubly standardized log response times (Fekken and Holden, 1992). Each log response time is centered twice by first subtracting the average log response time in a question and then by subtracting the average log response time of an examinee. This removes the time demand of an item and the speed of an examinee. The centered response times are then divided by the standard deviation. The Z^2^ value of an examinee is the sum of the squared doubly standardized log response times over the questions. The Z^2^ statistic is closely related to the log-normal model of van der Linden (2006) and very similar to the statistics proposed by Marianti et al. (2014) and Sinharay (2018). Although the three statistics differ in the way they weight the response times, they are highly correlated. In the present study, for example, their intercorrelation was *r* = 0.99. The present variant of Z^2^ has the advantage that it can be calculated without having to fit a latent trait model. As the doubly standardized log response times can informally be interpreted as standard normal random variates when the response times are log-normally distributed, a Z^2^ value close to the number of questions is assumed to be regular. Values exceeding this number considerably are indicative of data irregularities.

### 2.3. Indicators Based on Response Revisions

#### 2.3.1. N1 Statistic/NC1 Statistic

The N1 and NC1 statistic quantify how often an examinee changes his/her response during the test. They are based on the response in the last attempt. For each question *g*, it is recorded whether the response was revised by an examinee [N1(*g*) = 1] and whether this revision consisted in a change from the incorrect to the correct response [NC1(*g*) = 1]. The response sequences [100] and [011] generated during three attempts would, for example, be scored as N1(*g*) = 0 and NC1(*g*) = 0. The response sequence [010] would be scored as N1(*g*) = 1 and NC1(*g*) = 0. The response sequence [001] would be scored as N1(*g*) = 1 and NC1(*g*) = 1. Response sequences consisting of just one response are scored as N1(*g*) = 0 and NC1(*g*) = 0. The scores of an examinee in all questions are summed up. This yields the statistics N1 and NC1. High values of N1 and NC1 are unusual and may indicate cheating.

#### 2.3.2. N2 Statistic/NC2 Statistic

The N2 and NC2 statistics also quantify the number of revisions. In contrast to the N1 and NC1 statistic, they are based on the last revision of a question. Further attempts without a revision of the response are ignored. For each question *g* it is recorded whether an examinee changes his/her response at all [N2(*g*) = 1] and whether the last revision was a change from the incorrect to the correct response [NC2(*g*) = 1]. The response sequence [100] generated during three attempts would, for example, be scored as N2(*g*) = 1 and NC2(*g*) = 0. The response sequence [011], on the other hand, would be scored as N2(*g*) = 1 and NC2(*g*) = 1. Note that in both sequences, the last revision occurred during the second attempt. The response sequence [010] and [001] would be scored as the N1(*g*) and the NC1(*g*) statistic. Response sequences consisting of just one response are scored as N2(*g*) = 0 and NC2(*g*) = 0. The statistics N2 and NC2 are the sum of the scores of an examinee in the questions. High values of N2 and NC2 are supposed to indicate cheating. We consider the N2 and NC2 statistics as more indicative of cheating than the N1 and NC1 statistics. An illegitimately acquired response is usually assumed to be correct and will not be changed during the exam anymore. Furthermore, the N2(*g*) statistic will not be inflated when examinees proofread their test.

#### 2.3.3. T1 Statistic/T2 Statistic/T3 Statistic

The T1, T2, and T3 statistic reflect the time that is needed for responding to a question. For these statistics, the response time in last revision of a response is considered. If for example, an examinee had the response sequence [100] and the corresponding response times [1.1, 0.8, 2.1], one would use the response time 0.8. This response time will be denoted as revision time T1(*g*) in the following. In case there was no revision, the first response time is used. The revision times T1(*g*) are the basis for three statistics. The first statistic T1 is simply the sum of the revision times T1(*g*) over the question. The second statistic is based on the relative revision times in the questions, which are defined as the ratio of the revision times T1(*g*) and the response times in the first attempt. The relative revision time would, for example, be 0.8/1.1 for the response sequence described above. The second statistic T2 is the sum of the relative revision times over the times. The third statistic T3 is also based on the relative revision times. It is defined as the interquartile range of the relative revision times of an examinee. In contrast to T1 and T2 that reflect the level of the revision times, the statistic T3 captures changes in work pace within an examinee. As revisions due to test collusion are faster than regular responses, we assume that T1 and T2 have low values in cheaters. T2 is an improvement over T1 as it takes account of the general response speed of an examinee. T3 is motivated by the conjecture that response times vary widely when cheating times are mixed with regular response times. A large value of T3 is therefore indicative of partial cheating.

### 2.4. Indicators Based on the Speed-Accuracy-Relation

#### 2.4.1. CD Statistic

The CD statistic assess whether an examinee's data pattern is consistent with the general relation between the test score and the total testing time in the sample. The CD statistic is defined as Cook's distance of an examinee in the regression of the total testing time on the test score. Cook's distance is a measure of the influence an observation has on the parameters of a regression model. It assesses whether the model's predictions change when an observation is deleted. The distance is large when an observation is an outlier with respect to the predictor (leverage point) and has a large residual. Large values of the CD statistic are considered as indicative of data irregularities. Note that a similar measure was used by Engelhardt and Goldhammer (2019) for the validation of tests.

### 2.5. Indicators Based on the Number of Identical Responses

#### 2.5.1. PT Statistic/PI Statistic

The PT and PI statistic assess how similar the response patterns of different examinees are. For the PT statistic, one matches each response pattern to the response pattern in the sample that is most similar. This is the response pattern that has the highest number of identical responses. The PT statistic is the relative frequency of identical responses in the response pattern and its match. The PI statistic is similar to the PT statistic with one exception. In order to determine the most similar response pattern, only the incorrect responses are considered. The PI statistic is the relative frequency of identical incorrect responses in the response pattern and its match. For the PT and PI statistic, the final response is used. The PT and PI statistic have high values in examinees that copy responses from other examinees. The PT statistic, however, will also have a high value in examinees with a high test score as these examinees will necessarily have similar response pattern.

## 3. Method

The objective of the study was 3-fold. First, we aimed at evaluating the power of the indicators to separate regular respondents from cheaters. Furthermore, we aimed at assessing the relative importance of the indicators for the detection of cheating. Finally, we wanted to determine the detection rate that can be achieved by using all indicators jointly. We approached the problem of cheating detection experimentally. The experimental setting was a mock exam conducted at a university. Some examinees were instructed to cheat such that the cheaters were known beforehand. By this proceeding, we were able to use field data (high ecological validity) and could still assess the true detection rate. This complements pure simulation studies, where the data are perfectly model conform and results depend on artificial simulation conditions, and pure empirical studies, where cheaters are not certainly known.

### 3.1. Participants

Data were collected in 01/2019 (Time 1) and 01/2020 (Time 2). Participants were recruited from the cohort of education undergraduate students who were enrolled in an introductory lecture on psychology at a university. All registered students in the lecture were invited to take part in a mock exam. The mock exam was scheduled 1 week before the final exam the students had to take in order to pass the psychology module. The students were told that the mock exam would be similar to the regular exam and could be used as self-assessment. The students were also informed that participation was entirely voluntary, that their results would not count toward their final grade, and that there would be no disadvantages for those choosing not to participate. In 01/2019, 971 students were registered in the lecture. All students were invited to take the mock exam, but only 325 students participated. The data of 16 students were not recorded properly and had to be excluded. Data from further five students had to be excluded as the students did not finish the exam. The data of the remaining 304 students were used for data analysis (76% female; on average 21.3 years of age, min = 19; max = 42). Two hundred and seventy students were assigned to the reference condition. These students formed the reference group of regular responders. The remaining 34 students were assigned to two experimental conditions. These students formed the experimental groups of cheaters (Condition 1: 12 students, Condition 2: 22 students). In 01/2020, 834 students were registered in the lecture and invited to take the mock exam. This time, 460 students participated. Data of 25 students had to be excluded due to technical problems. Further six students were excluded as they did not finish the exam regularly or did not follow the instructions. The data of the remaining 429 students were used for data analysis (76% female; on average 21.4 years of age, min = 18; max = 48). 397 students were assigned to the reference condition. These students formed the reference group of regular responders. The remaining 32 students were assigned to two experimental conditions. These students formed the experimental groups of cheaters (Condition 1: 15 students, Condition 2: 17 students). The combined sample had a total sample size of 733 students, 667 in the reference group and 66 in the experimental groups.

### 3.2. Materials

Data were collected on the mock exam and further variables. These variables included motivational scales (Knekta and Eklöf, 2015), additional short cognitive tests and demographics (e.g., gender or age). As the scales and the additional tests are not relevant for the present paper, we will not describe them in detail. Interested readers are invited to contact the authors for more information.

The mock exam was computer assisted. At the beginning of the exam, the students were shown a list of keywords that each referred to a question. Students could choose a keyword from the list. The full question was then presented on the screen. The questions consisted of an introduction and four response options, of which the correct response had to be chosen (multiple choice format). Students could either select a response option or decide to abort responding. In either case they returned to the list, from which they could choose the next question. When a question was chosen, the time was recorded from the onset of the question till the return to the list. Examinees were not restricted in the way they worked on the test. Questions that had already been processed could be selected again. There was no fixed question order and no time limit. Paper based notes were not allowed. The progress was indicated by highlighting all questions to which the examinee already had responded. An examinee could end the exam by pressing 000 and Enter. At the end of the exam, each examinee was informed about his/her performance. With this implementation of the exam, we intended to allow the examinees a similar control over their proceeding as in a paper and pencil test; see the Pisa-based test for schools for a similar proceeding (Wise and Gao, 2017).

The mock exam was identical at Time 1 and Time 2 and consisted of 20 questions. The questions were similar to the questions of the final exam each student had to take in order to pass the psychological module 1 week later. Some questions were simple recall questions. These questions, for example, asked for the name of a scholar and his/her psychological theory. Other questions were more complex and required the prediction of experimental results. For each question call, the chosen response option and the time from the presentation till the response/decision to abort were stored. We also recorded the sequence the students worked on the questions.

### 3.3. Procedure

The mock exam took place in 01/2019 and 01/2020 at the end of each lecture. The mock exam was scheduled 1 week before the final exam. All students registered for the lecture received an e-mail via the university's intranet. The e-mail contained a link to a web page where the students could register for the mock exam. In the e-mail, we also asked for the participation in one of two additional accompanying studies about learning strategies that would take place immediately after the mock exam. The participation in the accompanying studies would be compensated by a monetary reward (Study I: 8€/ Study II: 16€). The students were told to contact a student assistant in case they were interested in participating in the mock exam and one of the studies. In this way, we recruited 34 students—12 for the first study and 22 for the second study—in 01/2019 and 32 students—15 for the first study and 17 for the second study—in 01/2020.

The participants in the additional studies were actually recruited in order to induce cheating. Depending on the date of the mock exam and the additional study the students were recruited for, the cheating manipulation differed. Students recruited for the first study (Condition 1) were involved in a form of test collusion. Students recruited for the second study (Condition 2) were provided with pre-knowledge. The form and amount of cheating varied from 2019 (Time 1) to 2020 (Time 2). Fully crossing two cheating strategies (test collusion, pre-knowledge) with two amounts of cheating (Time 1, Time 2) resulted in the following four experimental groups:

#### 3.3.1. Group TT1 (Time 1/Condition 1)

The participants were informed that the study was in fact about cheating on tests. They were told to leave the room during the mock exam about 10 min after beginning the examination. Outside the room, they would receive a small note containing the correct responses to the questions from a fictitious fellow student. Having returned, their job would be to use the note without being caught. The note contained the correct solutions to all questions. It was handwritten and mimicked the typical note one student would pass to another during the test. In this way, we simulated cheating in the form that a student receives the correct responses to all questions by an insider, an extreme form of test collusion (e.g., Belov, 2013). Although we aimed at 20 participants in order to achieve a cheating rate of 7% as reported by McCabe (2016), we only could recruit 12.

#### 3.3.2. Group TT2 (Time 2/Condition 1)

The participants received a similar instruction at Time 2 as at Time 1. They were told that they would receive a note containing the responses of a peer student about 15 min after they had started the exam. They should use the note during the exam without being caught. The note contained the real responses of an examinee from Time 1. Although the examinees, whose responses were used, were chosen from the upper half of the students, not all their responses were correct. The notes were handwritten. In this way, we simulated cheating in the form that an examinee collaborates with another by passing a solution sheet with all his/her actual responses, a milder form of test collusion. We aimed at 20 participants, but could only recruit 15.

#### 3.3.3. Group PN1 (Time 1/Condition 2)

Participants were not informed about the true aim of the study. They were told that the study was about learning strategies. They were required to attend a course 1 day before the mock exam. In this course, a list of eight practice questions was passed out and the student were invited to work on them. The students were informed that the questions of the mock exam would be similar to the practice questions and that we intended to assess whether familiarity with the response format improves the performance in the test. After working on the practice questions, the participants received the correct responses. The eight practice questions of the list were all included in the mock exam the next day. This corresponds to 40% (8/20) possible pre-knowledge with regard to the questions of the mock exam. When composing the list, we tried to equilibrate the leaked and new questions with respect to their time demand and difficulty. As the properties of the questions were unaware to us at Time 1, we used word counts and expert ratings for this purpose.

#### 3.3.4. Group PN2 (Time 2/Condition 2)

Participants received the same cover story at Time 2 as at Time 1. They were informed about the alleged aim of the study and were requested to take part in a course 1 day before the mock exam. In the course, the participants received a list with sixteen practice questions. They were told that the questions were taken from the actual question pool and that, by some chance, one or more questions could be selected for the final exam. The participants were invited to work on the questions and received the correct responses at the end. Only eight of the sixteen practice questions were included in the mock exam the next day. This corresponds to 40% (8/20) possible pre-knowledge with regard to the questions of the mock. The leaked questions at Time 2 were different from the leaked questions at Time 1. We interchanged some questions in order to increase the similarity between the leaked and the new questions. In doing so, we used item statistics from Time 1.

The mock exam was conducted in a lecture hall where all computer assisted examinations of the university took place. Students were tested blockwise in sessions of up to 80 students. Although the exam was low stakes—such that there should be little motivation to cheat—we took precautions to prevent all forms of cheating behavior that were not induced. The mock exam was proctored by four instructors. Examinees were spread out and randomly assigned to a seat. We randomized the assessment order (mock exam first vs. cognitive tests first) to hamper not intended and not induced answer copying by looking at the screens of other students. At the end of the mock exam, we took care that the students could not talk to their immediate successors. We also instructed the students not to talk about the exam with any other students who did not have taken it yet.

All participants were treated in accordance with the ethical guidelines of the American Psychological Association. Data were collected and stored anonymously. No data file could be associated with a specific participant.

### 3.4. Analysis

We exclusively analyzed the data of the mock exam. The data of the final exam were not considered. We first analyzed whether the data from Time 1 and Time 2 differed in the students who did not participate in Condition 1 or Condition 2 (in the following referred to as regular respondents). As there were hardly any differences in the distribution of the responses, the response times, the number of revisions and the frequency of identical responses, we decided to pool the data for all subsequent analyses. The indicators were calculated as described above. The indicators based on the responses (U1, U3, CS, H^T^) were determined with the final response, the indicators based on the response times (KL, KT, Z^2^) with the total time spent on a question, that is, the sum of the times in all attempts. We additionally considered the total testing time (ST) over all items (Meade and Craig, 2012) that served as a benchmark for the more complex indicators.

Before analyzing the data further, we screened the data for rapid guessers and checked the test taking motivation with the motivational scales. We then calculated simple descriptive statistics, estimated the internal consistency of the responses and response times, calculated the indicators of cheating as described above and assessed their interrelations. Then, we analyzed whether the reference group and the different experimental groups differed in the levels of the indicators. We distinguished five groups: The examinees responding regularly from the reference group (RR), the colluding examinees in Condition 1 at Time 1 (TT1), the colluding examinees in Condition 1 at Time 2 (TT2), the examinees with pre-knowledge in Condition 2 at Time 1 (PN1) and the examinees with pre-knowledge in Condition 2 at Time 2 (PN2); see the previous paragraph for a description of the groups. Group differences were investigated with an analysis of variance and the effect size η^2^ (Cohen, 1973). We conducted two different analyses. In the first analysis, we included the RR examinees and all groups of cheaters (PN1, PN2, TT1, TT2) at the same time. As the effect size η^2^ depends on the case numbers, we weighted the examinees in order to counterbalance the different subsample sizes in the cheating conditions. We mimicked the case of 80% RR examinees, 5% PN1 examinees, 5% PN2 examinees, 5% TT1 examinees, and 5% TT2 examinees. In the second analysis, we contrasted the RR examinees with each group of cheaters separately. For sake of comparability, we weighted the examinees in order to achieve a relation of 90% RR examinees and 10% cheaters. The weights conform to the occurrence of cheating in the US (Bernardi et al., 2008; McCabe, 2016). We also investigated the separability of the groups graphically by means of receiver operating characteristic (ROC) curves. ROC curves depict the true positive rate against the false positive rate at various decision thresholds.

Finally, we employed a classification tree in order to detect the cheaters (Breiman et al., 1984). The usage of data mining techniques for the detection of cheating has been popularized recently (e.g., Burlak et al., 2006; Kim et al., 2017; Toton and Maynes, 2018; Man et al., 2019; Zopluoglu, 2019). We chose a classification tree as the procedure generates an easily communicated rule for the detection of cheaters. A classification tree also provides cut-points and is capable to combine several indicators in a non-compensatory way. The classification tree was determined with the rpart package (Therneau and Atkinson, 2019) within the software environment R (R Development Core Team, 2009). The standard implementation was used (Gini homogeneity, 0/1 loss). All indicators were entered and the tree was grown until no improvement could be achieved or the number of examinees within the subgroups fell below 5. The full tree was then pruned and the relative importances of the indicators were determined. The relative importance of a variable sums the contribution of an indicator to the purity of each node at each split where the indicator is used plus the splits where the indicator is a surrogate. It is a normed measure lying within the range from 0 to 100; see Therneau and Atkinson (2019) for more details. We fit two different classification trees. A first classification tree was grown in order to separate the RR examinees from the cheaters. For this analysis, the four groups of cheaters were merged into one. A second classification tree was grown in order to separate all five groups of examinees (RR, PN1, PN2, TT1, TT2). When fitting the classification trees, we employed the weights reported above (Elkan, 2001).

## 4. Results

### 4.1. Descriptive Statistics

A visual inspection of the response time distributions suggested that rapid guessing was absent in the data. This finding was corroborated by the fact that the self-assessments of the test taking motivation on the motivational scales (effort, importance) was above the center point of the rating scale in most participants. Descriptive statistics with respect to the average solution frequency ($\bar{x}$), the average processing time ($\bar{t}$), the average frequency each question was worked on ($\bar{v}$) and the average number of revisions ($\bar{c}$) are reported in Table 1 separately for Time 1 and Time 2. The statistics have been averaged over the questions except the number of revisions which refers to the whole exam. Results are reported for all 20 questions (All), for the eight leaked questions (Old) and for the remaining 12 questions that were new for all examinees (New); note that the leaked questions differed between Time 1 and Time 2. Results are given for the five groups of examinees.

**Table 1:** Sample size (n), average solution frequency ($\bar{x}$), average processing time ($\bar{t}$), average number of attempts ($\bar{v}$), and average number of revisions ($\bar{c}$) for all examinees and for the different groups differentiated according to the time of data collection.

**Quantity**	**Items**	**Time 1**				**Time 2**			
		**Total**	**RR**	**PN1**	**TT1**	**Total**	**RR**	**PN2**	**TT2**
*n*	–	304	270	22	12	429	397	17	15
$\bar{x}$	All	0.58	0.55	0.72	0.95	0.58	0.57	0.75	0.66
	New	0.61	0.60	0.58	0.94	0.59	0.59	0.60	0.63
	Old	0.54	0.49	0.93	0.96	0.56	0.53	0.97	0.70
$\bar{t}$	All	32.45	32.69	26.06	38.78	31.80	32.11	25.88	30.33
	New	25.28	24.86	28.07	29.59	32.93	32.86	34.98	32.52
	Old	43.21	44.44	23.03	52.56	30.10	30.98	12.23	27.05
$\bar{v}$	All	1.31	1.27	1.26	2.23	1.35	1.33	1.46	1.75
$\bar{c}$	All	1.10	0.84	1.32	6.58	0.90	0.79	0.76	3.93

*Results for $\bar{x}$, $\bar{t}$ and $\bar{v}$ have been averaged over the questions. Results are given for all questions (All), the eight questions affected by pre-knowledge (Old) and the 12 questions not affected by pre-knowledge (New). Total denotes all examinees, RR the regular respondents, PN the examinees with pre-knowledge and TT the examinees engaged in test collusion*.

The mock exam was rather difficult. The solution frequencies of the questions were around 0.58. None of the RR respondents could solve all questions. The distribution of the responses and response times at Time 1 and Time 2 were very similar in the RR examinees; see the statistics in the row *All* column *RR*. We therefore combined the data from Time 1 and Time 2. We assessed the internal consistency via McDonald's ω_*T*_ coefficient (Zinbarg et al., 2005). The internal consistency of the responses was ω_*T*_ = 0.61 in the data of the RR examinees, and thus modest according to common standards (Lance et al., 2006). The internal consistency of the response times was ω_*T*_ = 0.85. This indicates the presence of a general speed factor that exerts its influence irrespective of the content of the exam. Table 1 demonstrates that the experimental manipulation was effective. The average solution frequencies of the PN1 examinees (0.72) and the PN2 examinees (0.75) were higher than the average solution frequency of the RR examinees (Time 1: 0.55, Time 2: 0.57). The average response times of the PN1 examinees (26.06) and the PN2 examinees (25.88) were lower than the average response times of the RR examinees (Time 1: 32.69, Time 2: 32.11). The effect, however, was limited to the leaked items (Row *Old*). In the new items (Row *New*), the average solution frequencies and average response times were similar for the RR examinees, the PN1 examinees and the PN2 examinees. At Time 1, the leaked questions (Row *Old*) were more difficult and time demanding than the new questions (Row *New*). At Time 2, the leaked questions (Row *Old*) were more similar to the new questions (Row *New*) with respect to difficulty and time demand.

The solution frequencies of the TT1 examinees (0.95) and TT2 examinees (0.66) involved in test collusion were higher than the solution frequencies of the RR examinees (Time 1: 0.55, Time 2: 0.57). Somewhat unexpected, the average response time of the TT1 examinees (38.78) was higher than the average response time of the RR examinees (32.69). This might have been caused by the additional effort of revising questions that had been answered before the note was received; note that the TT1 examinees also made more attempts and more revisions than the RR examinees. The effect of test collusion on the solution frequency was less pronounced at Time 2 than at Time 1. This is due to the fact that the note used at Time 2 also contained incorrect responses. RR examinees worked on each question about 1.3 times on average. This implies that not all students did proofread their answers on the exam. In that case, each question would have been attempted at least twice.

The Pearson product moment correlations between the different indicators are given in Table 2. Results are reported for the RR examinees as the presence of cheaters would inflate the correlations. The indicators based on the responses all correlate highly. This is hardly surprising as they all assess the Guttman homogeneity of the response patterns. The indicators tapping the numbers of revisions are also strongly interrelated. Correlations are lower for the indicators based on the response times and the revision times. In general, the indicators that are based on the same quantity tend to correlate higher than indicators based on different quantities. A principal component analysis of the correlation matrix did not reveal a simple and interpretable way to summarize the data.

**Table 2:** Product moment correlation coefficients between the different indicators.

	**ST**	**KL**	**Z^2^**	**KT**	**U1**	**H^T^**	**U3**	**CS**	**N1**	**NC1**	**N2**	**NC2**	**T1**	**T2**	**T3**	**CD**	**PT**	**PI**
ST	1.00	−0.07	−0.02	−0.24	0.15	−0.15	0.16	0.16	0.21	0.17	0.28	0.23	0.78	0.02	0.06	0.32	−0.20	−0.17
KL	−0.07	1.00	0.96	−0.37	−0.04	0.06	−0.04	−0.05	0.15	0.17	0.18	0.21	−0.02	0.10	0.01	0.06	0.12	0.13
Z^2^	−0.02	0.96	1.00	−0.42	−0.04	0.05	−0.04	−0.04	0.13	0.16	0.18	0.21	0.03	0.11	−0.01	0.07	0.11	0.10
KT	−0.24	−0.37	−0.42	1.00	−0.18	0.19	−0.18	−0.19	−0.07	−0.06	−0.12	−0.09	−0.16	−0.04	−0.02	−0.05	0.11	0.11
U1	0.15	−0.04	−0.04	−0.18	1.00	−0.96	0.94	0.97	0.06	0.05	0.07	0.06	0.15	−0.02	0.01	0.08	−0.43	−0.28
H^T^	−0.15	0.06	0.05	0.19	−0.96	1.00	−0.92	−0.96	−0.04	−0.06	−0.05	−0.06	−0.16	0.01	0.02	−0.05	0.38	0.27
U3	0.16	−0.04	−0.04	−0.18	0.94	−0.92	1.00	0.98	0.06	0.05	0.08	0.06	0.16	−0.03	0.03	0.10	−0.46	−0.31
CS	0.16	−0.05	−0.04	−0.19	0.97	−0.96	0.98	1.00	0.06	0.06	0.08	0.07	0.16	−0.02	0.02	0.09	−0.42	−0.27
N1	0.21	0.15	0.13	−0.07	0.06	−0.04	0.06	0.06	1.00	0.87	0.90	0.81	−0.12	0.10	0.51	0.13	−0.05	−0.03
NC1	0.17	0.17	0.16	−0.06	0.05	−0.06	0.05	0.06	0.87	1.00	0.79	0.92	−0.10	0.23	0.28	0.09	0.02	0.05
N2	0.28	0.18	0.18	−0.12	0.07	−0.05	0.08	0.08	0.90	0.79	1.00	0.87	−0.13	0.08	0.46	0.22	−0.03	−0.04
NC2	0.23	0.21	0.21	−0.09	0.06	−0.06	0.06	0.07	0.81	0.92	0.87	1.00	−0.11	0.21	0.25	0.17	0.06	0.05
T1	0.78	−0.02	0.03	−0.16	0.15	−0.16	0.16	0.16	−0.12	−0.10	−0.13	−0.11	1.00	−0.02	−0.06	0.30	−0.18	−0.15
T2	0.02	0.10	0.11	−0.04	−0.02	0.01	−0.03	−0.02	0.10	0.23	0.08	0.21	−0.02	1.00	0.04	−0.10	0.03	0.05
T3	0.06	0.01	−0.01	−0.02	0.01	0.02	0.03	0.02	0.51	0.28	0.46	0.25	−0.06	0.04	1.00	0.11	−0.10	−0.06
CD	0.32	0.06	0.07	−0.05	0.08	−0.05	0.10	0.09	0.13	0.09	0.22	0.17	0.30	−0.10	0.11	1.00	−0.10	−0.05
PT	−0.20	0.12	0.11	0.11	−0.43	0.38	−0.46	−0.42	−0.05	0.02	−0.03	0.06	−0.18	0.03	−0.10	−0.10	1.00	0.74
PI	−0.17	0.13	0.10	0.11	−0.28	0.27	−0.31	−0.27	−0.03	0.05	−0.04	0.05	−0.15	0.05	−0.06	−0.05	0.74	1.00

*Results for n = 667 regular respondents. ST, Total testing time; KL, Kullback-Leibler statistic; Z^2^, Squared doubly standardized RT; KT, Kendall's tau statistic; U1, U1 statistic; H^T^, H^T^ statistic; U3, U3 statistic; CS, Sato's C; N1, Revisions last attempt; NC1, Correct revisions last attempt; N2, Revisions last change; NC2, Correct revisions last change; T1, Time for last revisions; T2, Relative time for last revisions; T3, Interquartile range of relative revision times; CD, Cook's distance; PT, Maximal number of identical responses; PI, Maximal number of identical incorrect responses*.

### 4.2. Discriminatory Power

The discriminatory power of the indicators to separate the five groups of examinees (RR, PN1, PN2, TT1, TT2) was analyzed next. For this purpose, we used an analysis of variance model (ANOVA). In a first analysis, we analyzed the difference of the RR examinees to each group of cheaters separately. In doing so, we contrasted the RR examinees with each of the four groups of cheaters in four separate ANOVAs. These analyses served in order to assess whether the indicators can detect one specific form of cheating. In a second analysis, we analyzed the differences between all groups jointly. The purpose of this analysis was to investigate the overall performance of the indicators. In all analyses, the variance components of the ANOVA were used in order to determine the effect size η^2^. We do not report the *F* statistic and the *p*-value, because the assumptions of the ANOVA (normality, homoscedasticity) were not met and statistical significance does not imply practical significance. The effect size is reported for all indicators in Table 3. In addition to the effect size, the means and standard deviations of the indicators are given for the different groups of examinees.

**Table 3:** Means (m) and standard deviations (sd) of the indicators for the five groups as well as the effect sizes η^2^ for the group specific analysis and the analysis where the groups were considered jointly.

**Ind**	**Basis**	**Group**														
		**RR**		**PN1**			**PN2**			**TT1**			**TT2**			**ALL**
		**m**	**sd**	**m**	**sd**	**η^2^**	**m**	**sd**	**η^2^**	**m**	**sd**	**η^2^**	**m**	**sd**	**η^2^**	**η^2^**
U1	X	0.26	0.12	0.45	0.13	0.18	0.28	0.15	0.00	0.15	0.27	0.05	0.19	0.09	0.03	0.13
H^T^	X	0.22	0.11	0.08	0.10	0.14	0.19	0.15	0.01	0.68	0.49	0.36	0.27	0.08	0.02	0.34
U3	X	0.22	0.12	0.30	0.11	0.03	0.33	0.14	0.06	0.10	0.18	0.08	0.20	0.10	0.00	0.10
CS	X	0.48	0.24	0.78	0.27	0.12	0.62	0.27	0.03	0.28	0.52	0.04	0.39	0.19	0.01	0.10
ST	T	64.59	6.38	59.83	5.24	0.05	56.51	8.67	0.12	68.53	6.67	0.03	63.23	6.31	0.00	0.11
KL	T	0.12	0.06	0.27	0.13	0.28	0.26	0.14	0.25	0.15	0.13	0.02	0.10	0.03	0.01	0.26
Z^2^	T	17.92	9.43	42.64	22.33	0.30	40.64	19.80	0.28	23.46	20.60	0.02	15.10	5.12	0.01	0.28
KT	T	0.47	0.14	0.04	0.24	0.42	0.30	0.11	0.12	0.41	0.13	0.02	0.51	0.11	0.01	0.33
N1	R	0.52	1.00	0.82	1.40	0.01	0.47	0.08	0.00	4.00	3.69	0.33	2.80	3.90	0.16	0.25
NC1	R	0.40	0.85	0.64	1.05	0.01	0.47	0.80	0.00	3.75	3.41	0.36	1.93	2.74	0.13	0.28
N2	R	0.70	1.18	1.09	1.74	0.01	0.76	1.03	0.00	6.25	3.11	0.56	3.33	4.65	0.16	0.38
NC2	R	0.52	0.97	0.86	1.36	0.01	0.71	0.92	0.00	6.00	2.86	0.62	2.33	3.18	0.14	0.46
T1	TR	548.22	168.06	431.16	146.51	0.04	392.56	153.96	0.07	405.01	160.95	0.06	418.47	99.55	0.05	0.10
T2	TR	20.02	1.30	19.79	0.79	0.00	20.30	1.00	0.00	16.97	2.39	0.28	18.74	3.41	0.05	0.18
T3	TR	0.00	0.04	0.00	0.01	0.00	0.00	0.00	0.00	0.27	0.30	0.39	0.19	0.34	0.20	0.30
CD	T/X	0.00	0.01	0.00	0.00	0.00	0.00	0.01	0.01	0.02	0.02	0.17	0.00	0.01	0.00	0.14
PT	X	0.75	0.08	0.81	0.61	0.04	0.83	0.05	0.09	0.96	0.07	0.40	0.86	0.08	0.14	0.33
PI	X	0.71	0.12	0.81	0.14	0.06	0.88	0.13	0.15	0.31	0.47	0.29	0.80	0.11	0.05	0.29

*Results for 733 examinees. ST, Total testing time; KL, Kullback-Leibler statistic; Z^2^, Squared doubly standardized RT; KT, Kendall's tau statistic; U1, U1 statistic; H^T^, H^T^ statistic; U3, U3 statistic; CS, Sato's C; N1, Revisions last attempt; NC1, Correct revisions last attempt; N2, Revisions last change; NC2, Correct revisions last change; T1, Time for last revisions; T2, Relative time for last revisions; T3, Interquartile range of relative revision times; CD, Cook's distance; PT, Maximal number of identical responses; PI, Maximal number of identical incorrect responses*.

The indicators that best discriminate the groups are the indicators based on the number of revisions. Indicator NC2, the number of wrong-to-right changes in the last revision, performs best (η^2^ = 0.46). Its mean is 0.52 in the RR examinees, but 2.86 in the TT1 examinees and 3.18 in the TT2 examinees. The indicators based on the response times are also quite powerful, the KT indicator being the best (η^2^ = 0.33). Its mean drops from 0.47 in RR examinees to 0.04 in PN1 examinees and to 0.11 in PN2 examinees. Findings for the indicators based on response times are mixed. The H^T^ statistic (η^2^ = 0.34) and the PT statistic (η^2^ = 0.33) perform well. Other indicators are less powerful. With respect to the different forms of cheating, there is no indicator that is powerful in all conditions. The indicators based on response time are capable to detect pre-knowledge (average η^2^ = 0.28), but not test collusion (average η^2^ = 0.02). The indicators based on the response revisions are capable to detect test collusion in the TT1 condition (average η^2^ = 0.47), but lack power to detect pre-knowledge (average η^2^ = 0.01). None of the indicators is capable to detect the milder form of test collusion (TT2). These findings suggest that a combination of different indicators might be needed to separate all groups of cheaters from the RR examinees.

**Figure 1:**
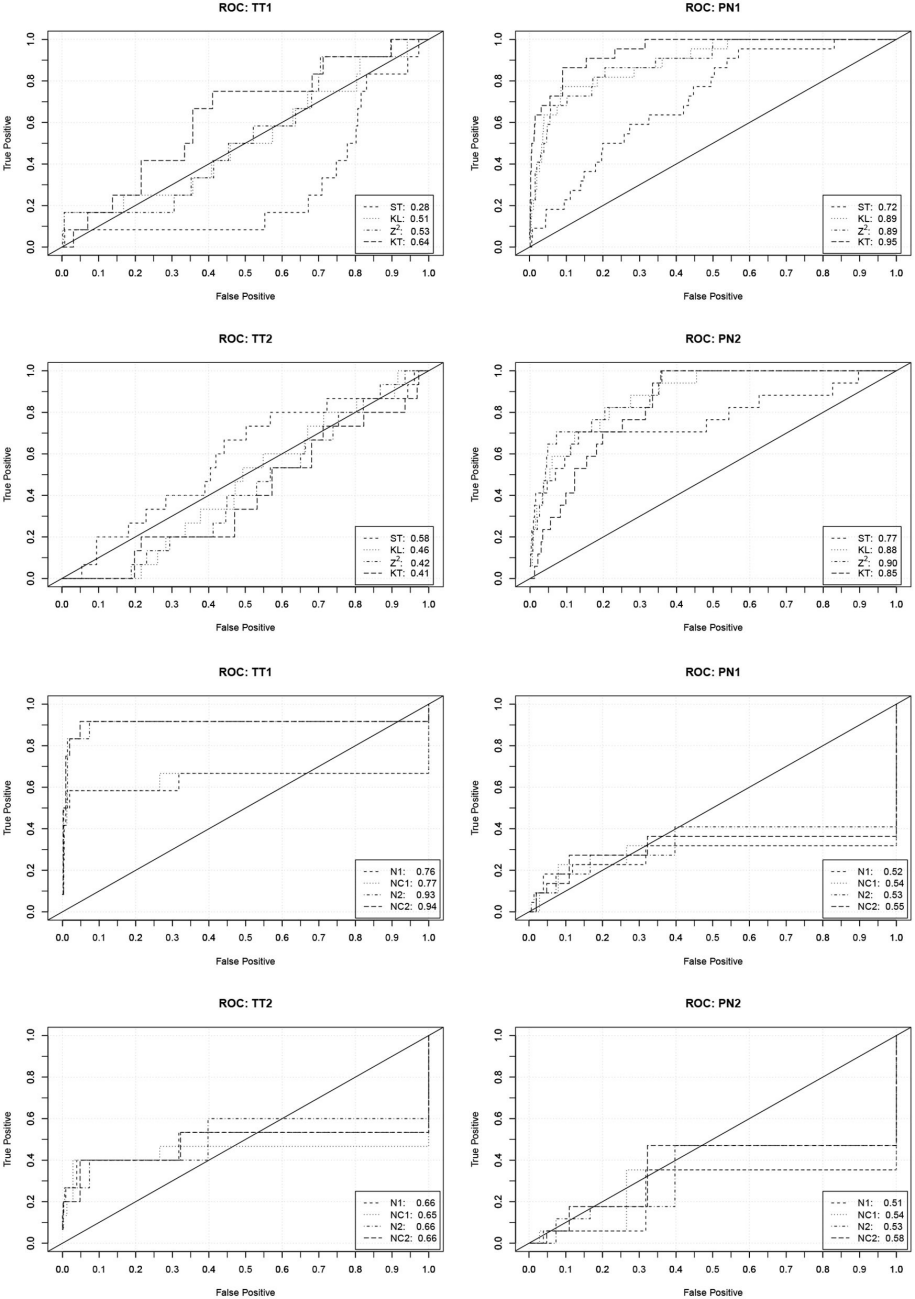
ROC curves (and area under the curve) describing the performance of the indicators based on the response times and the numbers of revisions to predict the four forms of cheating. TT1/TT2, Test collusion at Time 1/Time 2; PN1/PN2, Pre-knowledge at Time 1/Time 2.

**Figure 2:**
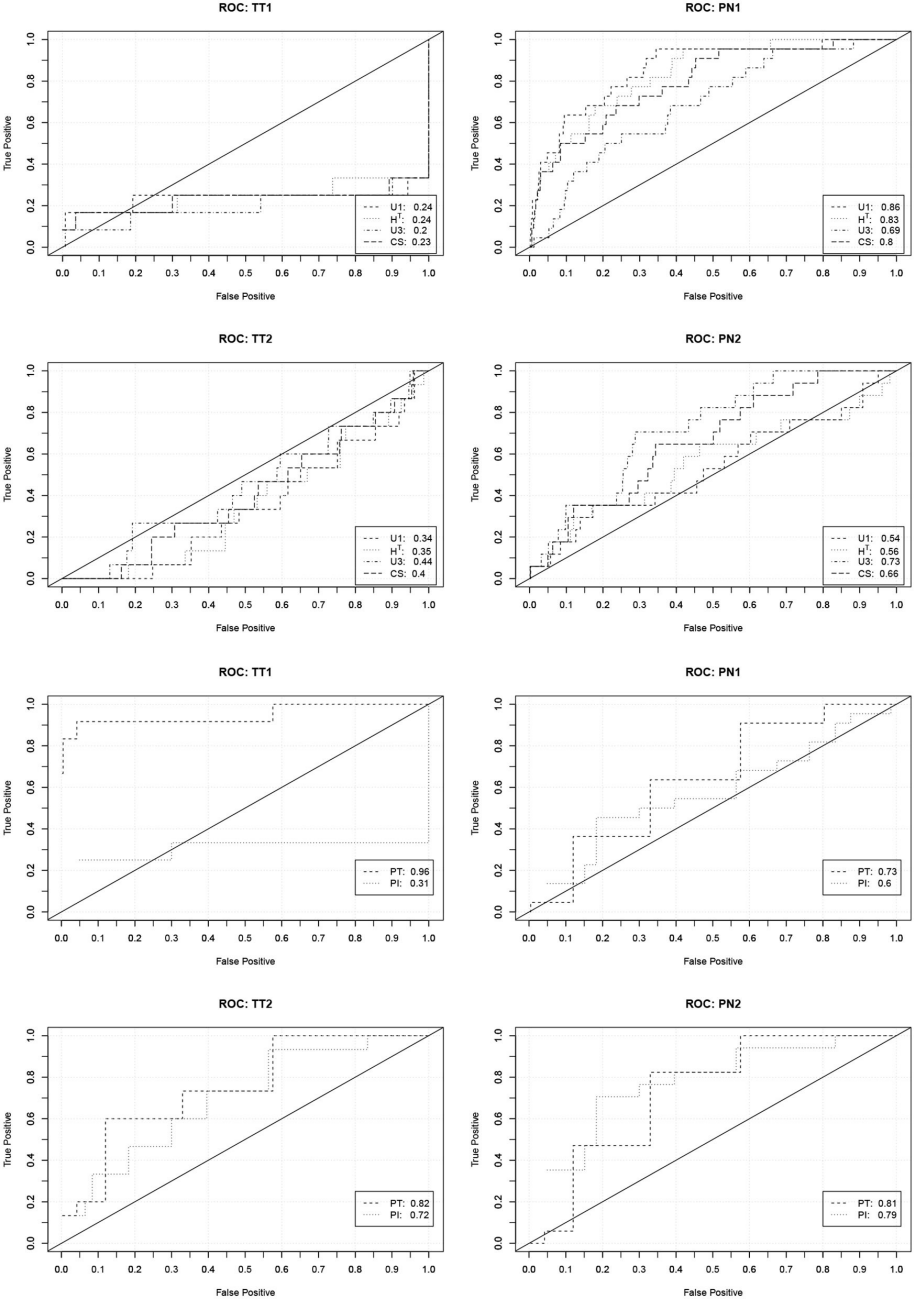
ROC curves (and area under the curve) describing the performance of the indicators based on the responses and the similarity of the response patterns to predict the four forms of cheating. TT1/TT2, Test collusion at Time 1/Time 2; PN1/PN2, Pre-knowledge at Time 1/Time 2.

In order to investigate the practical significance of the indicators, we determined ROC curves for the different forms of cheating. ROC curves depict how the true detection rate and the false alarm rate change when different values of an indicator are used as a decision criterion. A perfect indicator has a ROC curve that increases sharply from zero to one. A poor indicator has a ROC curve that falls below the diagonal. The area under the curve can be used as a performance measure. Figure 1 contains the ROC curves of the indicators based on the response times and number of revisions. Figure 2 contains the ROC curves of the indicators based on the responses and the similarity of the response patterns. Further ROC curves are not included in order to save space.

The curves corroborate the previous findings. The indicators based on the response times are only capable to detect pre-knowledge. The detection rates, however, are quite high in this case. With a false detection rate (Type-I error rate) of 0.05 about 60% of the PN1 examinees and about 40% of the PN2 examinees can be detected. The indicators based on the revisions only indicate the extreme form of test collusion. Their performance, however, is very good in this case. With a false detection rate of 0.05 over 80% of the TT1 examinees can be detected.

### 4.3. Classification Tree

Having evaluated the discriminatory power of the single indicators, we investigated their joint capability to separate the groups of examinees. For this purpose, we fitted two classification trees to the data. For the first tree, we merged the four groups of cheaters into one. The classification tree should simply separate the cheaters from the RR examinees. For the second tree, we distinguished the specific forms of cheating (PN1, PN2, TT1, TT2). The classification tree should predict the exact group the examinees were from. In both analyses, we excluded the indicators based on the responses and the indicators based the number of identical responses. As most TT1 examinees had solved all items, they had response patterns of perfect Guttman homogeneity and a high similarity. These cheaters were therefore easy to detect, by simply classifying all examinees with extreme values of the indicators as cheaters. Such a classification rule is problematic, as it will fail in easier exams where solving all questions is common. The technical details about fitting and implementation of the classification tree can be found in the methodology section; note that cases were weighted in order to equilibrate the different subsample sizes in the cheating conditions.

The first classification tree that separated the RR examinees from the cheaters had 41 splits. The full classification tree, however, overfits the data. A cross-validation suggested that the best classification tree was a pruned tree with six splits and seven end nodes. Three of the end nodes represented cheaters and four RR respondents. The importance of the variables in the pruned tree is given in Table 4, upper part. The N2 statistic was most important. The pruned classification tree classified 0.95 of the examinees in the original (unweighted) sample correctly. Altogether 28 of the 66 cheaters were detected (sensitivity: 0.42). Of the 667 RR examinees, seven were misclassified as cheaters (specificity: 0.99). When inspecting these cases, we could not identify a clear reason for the misclassification. All misclassified examinees provided irregular data with unusual values on several indicators. This implies that other irregular forms of test taking (e.g., occasional rapid guessing, pausing during the test) may appear as a form of cheating.

**Table 4:** Importance of indicators for the detection of cheating in the pruned classification tree.

**Tree 1: prediction of cheating**											
ST	KL	Z^2^	KT	N1	NC1	N2	NC2	T1	T2	T3	CD
6	6	9	6	10	8	16	12	2	11	12	2
**Tree 2: prediction of specific forms of cheating**											
ST	KL	Z^2^	KT	N1	NC1	N2	NC2	T1	T2	T3	CD
3	8	11	8	10	8	13	11	4	9	12	2

*Indicators based on the responses and number of identical responses have been excluded*.

**Figure 3:**
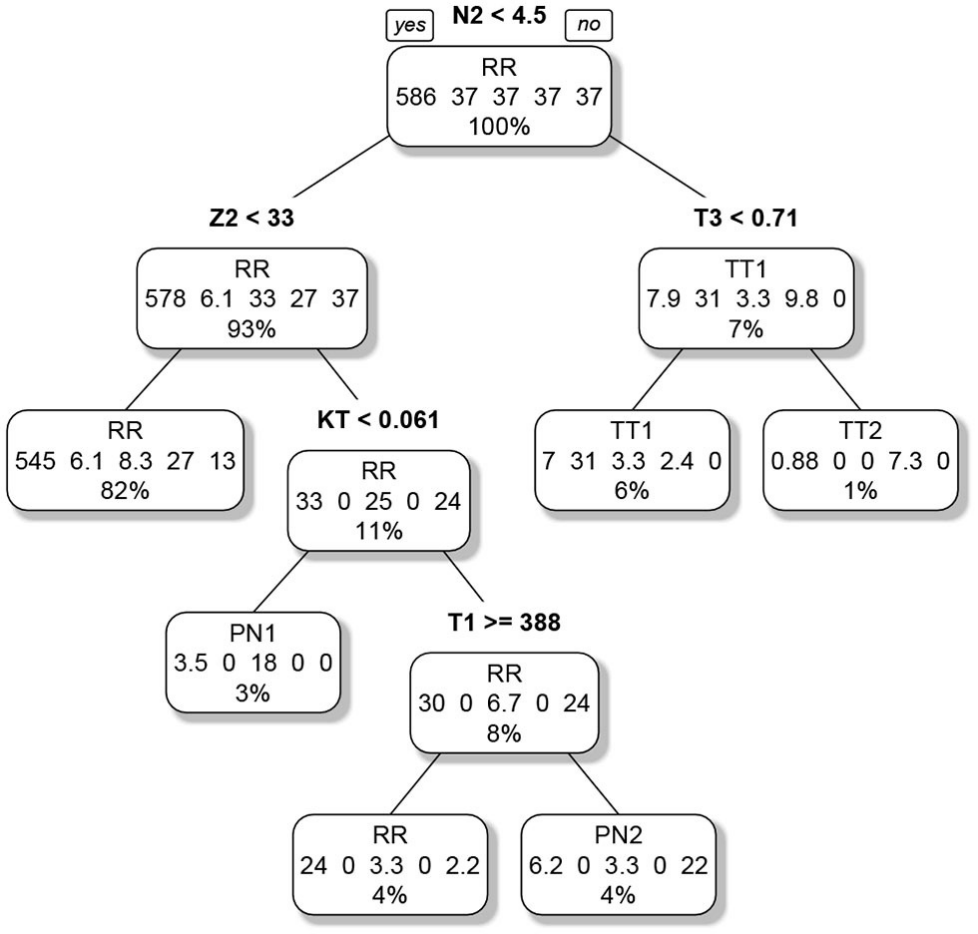
Pruned classification tree for the classification of the examinees into regular respondents and the four forms of cheating. The label of each node denotes the prediction. The numbers describe the composition of the node (RR/TT1/PN1/TT2/PN2). As the groups were weighted, the numbers do not correspond to the actual case numbers. The percentage informs about the relative number of examinees in each node in relation to the sample size of the analysis; note that RR denotes regular responding, TT1/TT2 test collusion at Time 1/Time 2 and PN1/PN2 pre-knowledge at Time 1/Time 2.

The second classification tree, which was trained to predict the actual group, had 45 splits. The tree was pruned in order to avoid overfitting. A cross-validation indicated that a pruned tree with five splits and six end nodes performed best. Two of the six end nodes represented RR respondents, the remaining four end nodes one form of cheating. The pruned classification tree is visualized in Figure 3. The importance of the variables is reported in Table 4, lower part. The N2 indicator was again most important. The pruned tree achieved an accuracy of 0.93 in the original (unweighted) sample. The first splits separate the general form of cheating (pre-knowledge/test collusion), the later eventually the specific variants (Time1/Time2).

## 5. Discussion

Cheating is a severe problem in educational testing and academic examination. Students who increase their test performance by illegitimate ways may surreptitiously obtain advantages they do not deserve. A high amount of cheating depreciates the value of academic achievement and creates a cheating culture that induces others to cheat (Rettinger and Kramer, 2009). Tolerating cheating is considered as unfair by the majority of students (Miller et al., 2015). Hence, considerable effort has to be undertaken to prevent and detect cheating. Unfortunately, recent technical inventions like micro cameras and wireless communication media have made cheating as easy as never before.

In the paper, we investigated whether two forms of cheating—test collusion and item pre-knowledge—are indicated by statistics that assess the regularity of an examinee's data. We considered these forms as they are probably the most serious forms of cheating. Basis of our investigation was a field study where cheating was induced experimentally. This approach had the advantage that the data were realistic on one hand and the cheaters were known on the other hand. High ecological validity is especially important when process data are used as in pure simulation studies the data often are unrealistically clean. In our study, the conditions for the detection of cheating were rather difficult. We used data from an exam that was—in contrast to the tests used in large scale assessment—not optimized with respect to its psychometric properties. The exam, for example, did not have a clear unidimensional dominance structure and its internal consistency (ω_*T*_ = 0.61) was slightly below the value 0.70 which is commonly considered as necessary for psychological research (Lance et al., 2006). This is a complication as several indicators of cheating implicitly assume that the data are from a scale with a unidimensional dominance structure (Guttman scale). When this assumption is violated, a Guttman pattern cannot be expected even in the test takers that behave regularly. Hence, the indicators might not perform as expected. The setting of the exam allowed for a high variability in test taking behavior. Examinees were allowed to revise responses, choose the question order freely, pause or even visit the bathroom during the exam. Hence, the data contained the whole range of test taking behavior one can observe in real life. This further impairs the statistics' capability to separate groups of test takers. In sum, there was no guarantee that the theoretical properties of the statistics would hold in practice.

The statistics that we considered as indicators of cheating were either based on the responses or on process data like the response times or the number of response revisions. When investigating the statistics' power to separate cheaters from regular respondents, it turned out that the indicators based on the process data (number of revisions and response times) performed best. This demonstrates the utility of process data for describing the students' test taking behavior. Nevertheless, the study also revealed that even the best indicators are not capable to separate regular respondents from cheaters perfectly. The natural variety in test taking behavior is so large that the group specific distributions of the indicators overlap considerably. Combining several indicators with a classification tree, for example, helps but only to a certain extent. This demonstrates the limitation of a statistical approach to the detection of cheating.

The current study is supposed to complement previous studies on cheating. We used real data and induced cheating artificially. Experimental studies of cheating are rare, the notable exceptions being studies by de Klerk et al. (2019) and Toton and Maynes (2019). Using real data has advantages but also limitations. First of all, the mock exam was low stakes, as it did not count to the final grade. Although it took place 1 week before the final examination and the students could use it as a self-test, their effort and amount of preparation was probably not as high as it would have been in a real exam. There was, however, no evidence for rapid guessing and the self-reported motivation was moderate to high. Announcing a price for high performers, as a reviewer suggested, might be a way to increase the motivation further in future studies. The exam was shorter than the typical exams used for course assessment. Whether this makes the detection easier or more difficult is hard to predict. The power of tests of person fit increases with the length of a test. Data from longer exams, however, might be more irregular due to fatigue or bad time management. This reduces the power. Although we were aware of these limitations, we could not investigate cheating in the real exam due to ethical reasons.

A second limitation is the fact that the results depend on the way we induced cheating. Some results depend on the distribution of the different forms of cheating in the sample. We counterbalanced this by weights. The weights were chosen in accordance with the frequency of cheating reported in the literature. The chosen weights, however, can be questioned and are not representative for all fields. The amount of cheating, for example, varies between business students and liberal arts students (Rettinger and Jordan, 2005). The form of cheating also impacts the findings. We simulated pre-knowledge and a form of test collusion. Whether our way of inducing pre-knowledge is representative for reality is difficult to evaluate. There are hardly any statistics on the amount and prevalence of pre-knowledge. It can be diffuse, consisting of unreliable records on previous questions but also be very precise in case the exam has been copied. General statements are difficult, as it depends on the amount of competition for grades whether students cooperate (share information) or not (Miller et al., 2015). The form of test collusion that we used in the study is also debatable. There are in fact two forms of answer copying. Examinees can copy answers on the fly, by simultaneously adopting an answer at the moment it is written down by their seatmate. We do not consider this form of copying as most relevant. Continuous online cheating will often be unsystematic and limited in its effects. It can also be prevented by screens and seating plans. In another form of answer copying, an examinee receives the responses to numerous questions all at once. Such cheating can not be online and has more impact on the test results. Whether such forms of massive answer copying are frequent, is hard to say. Surveys on cheating do not specify the way students copy from each other. Given the capacity of modern communication devices, one might speculate that massive forms of test collusion might become a problem in the future. More studies considering alternative forms of copying like partial string copying or random copying (Wollack, 2006) are needed.

A further limitation concerns the sample. The rate of non-response among the students was rather high. Only 33% of the registered students participated in the mock exam at Time 1 and only 55% of the students at Time 2. Whether this had an influence on the results is hard to appraise. As the participation rates differ between Time 1 and Time 2, the average test score and total testing however do not, the participation cannot be related to the test performance, provided that the distribution of ability and work pace in the population did not change. Whether there are systematic differences between participants and non-participants on other variables, is hard to assess. Furthermore, the sample size of the cheaters was rather low. Generalizations can therefore only be made with reserve. The cheaters might also not have been representative for the typical cheater, as cheating was instructed, not the student's decision. And last but not least, a cheater usually selects the person to copy from. This is probably a good student from his circle of friends.

In the study, we were capable to detect cheating on basis of the number of revisions and the response time pattern. The response pattern was of lesser importance. This suggests that process data may be more important for the detection of cheating than the classical statistics of item fit in some cases. Some word of caution is needed here, though. The number of revisions is only important when cheating involves that examinees change their responses during the exam. This happens only in case the examinees receive the information about the correct response with a delay; this may not be the case in some forms of cheating like the acquisition of an exam copy before taking the test or cheating on-the-fly. Cheating on-the-fly can probably best be detected by analyzing the similarity of response patterns between examinees and a similar pacing of the test (van der Linden, 2009; Maynes, 2017). Indicators based on the response times might also be limited in their capability to detect cheating. They require that cheating changes the time demand of the items differentially. Simply being somewhat faster in all questions or in the questions with lower time demand will not translate into an irregular response time pattern. It is the order of the response times that has to be changed by cheating. This is, in fact, not too different from the indicators based on the responses. A Guttman pattern will only be violated in case one unexpectedly solves some of the harder questions. Receiving the response to simple questions will not have much of an impact on the Guttman homogeneity. All these considerations imply that the classification rules that are represented by the two classification trees might not be transferable to other exams directly. An application to other exams might require an additional step of transfer learning whereby the distributions of the indicators from different samples are homogenized. This topic is currently under research.

## Data Availability Statement

The datasets presented in this study can be found in online repositories. The names of the repository/repositories and accession number(s) can be found below: The data sets [201906 irb-temea data.csv/201906 irb-temea.RData, 202002 irb-temea.2.RData] for this study are available on https://osf.io (doi: 10.17605/OSF.IO/NMXQ7).

## Ethics Statement

Ethical review and approval were not required for the study on adult human participants in accordance with the local legislation and institutional requirements. Adult volunteers provided their informed consent to participate in the current study. There was no deception. Materials and procedures were not invasive. Only anonymous data were collected.

## Author Contributions

All authors listed have made a substantial, direct and intellectual contribution to the work, and approved it for publication.

## Conflict of Interest

The authors declare that the research was conducted in the absence of any commercial or financial relationships that could be construed as a potential conflict of interest.
