# Supplementary material for: The Detection of Cheating on E-Exams in Higher Education—The Performance of Several Old and Some New Indicators
Source: Front Psychol. 2020 Oct 2;11:568825. doi: 10.3389/fpsyg.2020.568825 (PMC7573546; doi:10.3389/fpsyg.2020.568825)
Supplement: Supplementary file 1 [file Data_Sheet_1.pdf]

Supplementary Material

1 SUPPLEMENTARY DATA

Supplement A: Indicators Based on the Responses

This supplement contains details how the indicators based on the responses are determined. The following notation is used. The response of the $n = 1, ..., N$ examinees to the $g = 1, \ldots, G$ questions of the exam are denoted by x_{ng} and the response pattern of an examinee by $\boldsymbol{x}_n$. Responses are scored as either correct ($x_{ng} = 1$) or incorrect ($x_{ng} = 0$). The quantity $s(\boldsymbol{x}_n) = \sum_{g=1}^{G} x_{ng}$ represents the sum score. Solution frequencies are defined as $p_g = 1/N \cdot \sum_{n=1}^{N} x_{ng}$. Function $\mathrm{I}(x)$ is the indicator function. In the following, we assume that the questions have been ordered according to increasing difficulty, that is, $p_i > p_j$ for $i < j$.

U1 statistic. The U1 statistic of van der Flier (1977) weights the actual number of Guttman errors in a response pattern against the maximal number that could have occurred. It is defined by

$$\mathrm{U1}\big(\boldsymbol{x}_n\big) = \frac{\sum_{g=1}^{G-1} \sum_{h=g+1}^{G} \mathrm{I}\big(x_{ng} < x_{nh}\big)}{s\big(\boldsymbol{x}_n\big)\big(G - s(\boldsymbol{x}_n)\big)} \tag{S1}$$

for $0 < s(\boldsymbol{x}_n) < G$ and $\mathrm{U1}(\boldsymbol{x}_n) = 0$ otherwise. A value of zero indicates perfect Guttman homogeneity, a value of one the contrary.

U3 statistic. The U3 statistic of van der Flier (1982) is also based on the Guttman errors. It differs from U1 in that each error is penalized by the weight $w_g = \log(p_g/(1 - p_g))$. It is defined by

$$\mathrm{U3}\big(\boldsymbol{x}_n\big) = \frac{\sum_{g=1}^{s(\boldsymbol{x}_n)} w_g - \sum_{g=1}^{G} x_{ng}\, w_g}{\sum_{g=1}^{s(\boldsymbol{x}_n)} w_g - \sum_{g=G-s(\boldsymbol{x}_n)+1}^{G} w_g} \tag{S2}$$

for $0 < s(\boldsymbol{x}_n) < G$ and $\mathrm{U3}(\boldsymbol{x}_n) = 0$ otherwise. A value of zero indicates perfect Guttman homogeneity, a value of one the contrary.

CS statistic. The CS statistic proposed by Sato (1975) compares the covariance between the responses and the general solution frequencies with the covariance a perfect Guttman pattern would have. The CS statistic has a similar structure as the U3 statistic and is defined by

$$\mathrm{CS}\big(\boldsymbol{x}_n\big) = \frac{G \cdot \sum\limits_{g=1}^{s(\boldsymbol{x}_n)} p_g - G \cdot \sum\limits_{g=1}^{G} x_{ng}\, p_g}{G \cdot \sum\limits_{g=1}^{s(\boldsymbol{x}_n)} p_g - s(\boldsymbol{x}_n) \cdot \sum\limits_{g=1}^{G} p_g} \tag{S3}$$

for $0 < s(\boldsymbol{x}_n) < G$ and $\mathrm{CS}(\boldsymbol{x}_n) = 0$ otherwise. The case $\mathrm{CS} = 0$ indicates perfect Guttman homogeneity. The statistic has no fixed upper bound.

H^T statistic. The H^T statistic proposed by Sijtsma (1986) is the normed average covariance between a student's response pattern and the response patterns of all other students. It is defined by

$$\mathrm{H}^\mathrm{T}\big(\boldsymbol{x}_n\big) = \frac{1/(n-1) \cdot \sum\limits_{n \neq j} \mathrm{cov}(\boldsymbol{x_n}, \boldsymbol{x_j})}{1/(n-1) \cdot \sum\limits_{n \neq j} \max \mathrm{cov}(s(\boldsymbol{x}_n), s(\boldsymbol{x}_j))}. \tag{S4}$$

Here, the term $\max \mathrm{cov}(s(\boldsymbol{x}_n), s(\boldsymbol{x}_j))$ denotes the maximal covariance that can be achieved for two response patterns with sum score $s(\boldsymbol{x}_n)$ and $s(\boldsymbol{x}_j)$. This quantity is $\max \mathrm{cov}\big(s(\boldsymbol{x}_n), s(\boldsymbol{x}_j)\big) = \min\big(s(\boldsymbol{x}_n), s(\boldsymbol{x}_j)\big)\big(G - \max\big(s(\boldsymbol{x}_n), s(\boldsymbol{x}_j)\big)\big)/G^2$. The H^T statistic is zero when the average covariance between a student's response pattern and all other response patterns is zero. Its maximal value is one.

SUPPLEMENT B: INDICATORS BASED ON THE RESPONSES TIMES

This supplement contains details how the indicators based on the responses times are determined. The following notation is used. The response time of student n in question g is denoted as t_{ng} and his/her response time pattern as $\boldsymbol{t}_n$; the average response time in a question is denoted as $\bar{t}_g$ and the median response time as $\widetilde{t}_g$.

KL statistic. The KL statistic proposed by Man et al. (2018) evaluates the congruence of an individual response time profile with the average response time profile. It is based on the proportions $\mathrm{f}(t_{ng}) = t_{ng}/\sum\limits_{g=1}^{G} t_{ng}$ of the total testing time a student spends on question g. The proportions are compared with the proportions of the average response times $\mathrm{f}(\bar{t}_g) = \bar{t}_g/\sum\limits_{g=1}^{G} \bar{t}_g$ via the Kullback-Leibler divergence Kullback and Leibler (1951):

$$\mathrm{KL}\big(\boldsymbol{t}_n\big) = \sum_{g=1}^{G} \mathrm{f}(\bar{t}_g) \log\left(\frac{\mathrm{f}(\bar{t}_g)}{\mathrm{f}(t_{ng})}\right). \tag{S5}$$

The divergence is zero in case the two sets of proportions are identical. An unusual distribution of the total testing time over the items inflates the statistic.

KT statistic. The KT statistic assesses whether the ranking of the items with respect to the response times is similar to the ranking with respect to the typical response time. The agreement is assessed via Kendall's tau correlation between the individual response times and the median response times

$$\mathrm{KT}(\boldsymbol{t}_n) = \frac{\sum_{g=1}^{G-1}\sum_{h=g+1}^{G} \mathrm{I}\big(t_{ng} > t_{nh}, \widetilde{t}_g > \widetilde{t}_h\big) - \sum_{g=1}^{G-1}\sum_{h=g+1}^{G} \mathrm{I}\big(t_{ng} > t_{nh}, \widetilde{t}_g < \widetilde{t}_h\big)}{G \cdot (G-1)/2}. \tag{S6}$$

The KT statistic is one in case the order is identical.

Z^2 statistic. The Z^2 statistic of Marianti et al. (2014) is closely related to the lognormal model of van der Linden (2006). For sake of simplification, we propose a closely related alternative that can be defined without any reference to a response time model. Define by $\bar{t}^*_g - \bar{t}^*$ the time demand of a specific question on the log scale where $\bar{t}^*_g = 1/N \cdot \sum_{n=1}^{N} \log(t_{ng})$ and $\bar{t}^* = 1/(NG) \cdot \sum_{g=1}^{G}\sum_{n=1}^{N} \log(t_{ng})$. Define by $\bar{t}^*_n - \bar{t}^*$ the time demand of an examinee where $\bar{t}^*_n = 1/G \cdot \sum_{g=1}^{G} \log(t_{ng})$. The residual z_{ng} is the component of a log response time that is not accounted for by the time demand of the question and the examinee

$$z_{ng} = \log\big(t_{ng}\big) - \big((\bar{t}^*_n - \bar{t}^*) + (\bar{t}^*_g - \bar{t}^*) + \bar{t}^*\big). \tag{S7}$$

The Z^2_n statistic is just the sum of the standardized and squared residuals $\mathrm{Z}^2_n = \sum_{g=1}^{G} \big(z^2_{ng}/\mathrm{var}(z_{ng})\big)$, where $\mathrm{var}(z_{ng})$ is the residuals' variance in item g. Note the similarity to the variance decomposition in analysis of variance (ANOVA) models, the approach of Meijer and Sotaridona (2006), and the double standardization procedures of response times (e.g., Fekken and Holden, 1992). As the standardized residuals can informally be interpreted as standard normal variates when the response times are log-normally distributed, a value of Z^2 close to G is assumed to be regular.

SUPPLEMENT C: INDICATORS BASED ON THE SPEED-ACCURACY-RELATION

CD statistic. Cook's distance is a measure of the influence an observation has on the parameters of a regression model. It assesses whether the model's predictions change when an observation is deleted. The distance is large when an observation is an outlier with respect to the predictors (leverage point) and has a large regression residual. For the present purpose, Cook's distance was determined for all examinees after regressing the total testing time on the test score. With $s(\boldsymbol{t}_n)$ being the total testing time and $s(\boldsymbol{x}_n)$ the test score, the distance is defined by

$$\mathrm{CD}_n = \frac{h_{nn}}{1-h_{nn}} \frac{ze^2_n}{2}, \tag{S8}$$

where h_{nn} is the leverage with respect to $s(\boldsymbol{x}_n)$ and ze_n the studentized residual of the total testing time $s(\boldsymbol{t}_n)$; more details on Cook's distance can be found in any monograph on regression models such as Jobson (1991).

REFERENCES

Fekken, G. and Holden, R. (1992). Response latency evidence for viewing personality traits as schema indicators. *Journal of Research in Personality* 26, 103–120. https://doi.org/10.1016/0092-6566(92)90047-8

Jobson, J. (1991). *Applied Multivariate Data Analysis – Regression and Experimental Design* (New York: Springer). https://doi.org/10.1007/978-1-4612-0955-3

Kullback, S. and Leibler, R. (1951). On information and sufficiency. *Annals of Mathematical Statistics* 22, 79–86. https://doi.org/10.1214/aoms/1177729694

Man, K., Harring, J., Ouyang, Y., and Thomas, S. (2018). Response time based nonparametric Kullback-Leibler divergence measure for detecting aberrant test-taking behavior. *International Journal of Testing* 18, 155–177. https://doi.org/10.1080/15305058.2018.1429446

Marianti, S., Fox, J., Avetisyan, M., and Veldkamp, B. (2014). Testing for aberrant behavior in response time modeling. *Journal of Educational and Behavioral Statistics* 39, 426–451. https://doi.org/10.3102/1076998614559412

Meijer, R. and Sotaridona, L. (2006). *Detection of advance item knowledge using response times in computer adaptive testing*. LSAC research report series CT 03-03, Law School Admission Council, Newton, PA

Sato, T. (1975). *The construction and interpretation of S-P tables* (Tokyo: Meiji Tosho)

Sijtsma, K. (1986). A coefficient of deviance of response patterns. *Kwantitatieve Methoden* 7, 131–145

van der Flier, H. (1977). Environmental factors and deviant response patterns. In *Basic problems in cross-cultural psychology*, ed. Y. Poortinga (Amsterdam: Swets and Zeitlinger). 30–35

van der Flier, H. (1982). Deviant response patterns and comparability of test scores. *Journal of Cross-Cultural Psychology* 13, 267–298. https://doi.org/10.1177/0022002182013003001

van der Linden, W. (2006). A lognormal model for response times on test items. *Journal of Educational and Behavioral Statistics* 31, 181–204. https://doi.org/10.3102/10769986031002181
